# Quantitative analysis of persister fractions suggests different mechanisms of formation among environmental isolates of *E. coli*

**DOI:** 10.1186/1471-2180-13-25

**Published:** 2013-02-04

**Authors:** Niels Hofsteenge, Erik van Nimwegen, Olin K Silander

**Affiliations:** 1 Computational and Systems Biology, Biozentrum, University of Basel, Basel, Switzerland

**Keywords:** Persister, Antibiotic, Ampicillin, Ciprofloxacin, Nalidixic acid, Environmental isolates, *E. coli*

## Abstract

**Background:**

Bacterial persistence describes a phenomenon wherein a small subpopulation of cells is able to survive a challenge with high doses of an antibiotic (or other stressor) better than the majority of the population. Previous work has shown that cells that are in a dormant or slow-growing state are persistent to antibiotic treatment and that populations with higher fractions of dormant cells exhibit higher levels of persistence. These data suggest that a major determinant of the fraction of persisters within a population is the rate at which cells enter and exit from dormancy. However, it is not known whether there are physiological changes in addition to dormancy that influence persistence. Here, we use quantitative measurements of persister fractions in a set of environmental isolates of *E. coli* together with a mathematical model of persister formation to test whether a single general physiological change, such as cell dormancy, can explain the differences in persister phenotypes observed in different strains.

**Results:**

If a single physiological change (e.g. cell dormancy) underlies most persister phenotypes, then strains should exhibit characteristic fractions of persister cells: some strains will consistently have high fractions of persisters (dormant cells), whereas others will have low fractions. Although we found substantial variation in the fraction of persisters between different environmental isolates of *E. coli*, these fractions were not correlated across antibiotics. Some strains exhibited high persister fractions in one antibiotic, but low persister fractions in a second antibiotic. Surprisingly, no correlation in persister fractions was observed between any two drugs, even for antibiotics with nearly identical modes of action (ciprofloxacin and nalidixic acid).

**Conclusions:**

These data support the hypothesis that there is no single physiological change that determines the persistence level in a population of cells. Instead, the fraction of cells that survive antibiotic treatment (persist) depends critically on the specific antibiotic that is used, suggesting that physiological changes in addition to dormancy can underlie persister phenotypes.

## Background

Bacterial persistence is a form of phenotypic heterogeneity in which a subset of cells within an isogenic population is able to survive challenges with antibiotics or other stressors better than the bulk of the population [1]. The persistence phenotype is transient and non-genetic, in contrast to antibiotic resistance, which is due to genetic changes. However, the ability to form persister cells, or the fraction of persister cells that are present in a culture, can be genetically controlled (see below). The phenomenon of persistence has significant clinical relevance [2], and it may be a primary factor as to why many infections require long-course antibiotic treatment for successful resolution [3]. Indeed, many patients with chronic infections harbor pathogens with increased rates of persister formation [4]. Thus, one of the most important questions concerning persister formation is determining the mechanisms that allow cells to become physiologically recalcitrant to treatment with antibiotics or other stressors.

Recent work has suggested that persisters become drug tolerant because they enter a dormant or slow-growing state [5-9]. This dormant state is thought to protect them from the lethal action of antimicrobials, since many antibiotics interfere with proliferative processes, such as cell wall assembly, DNA replication, or protein synthesis [7,10].

Genetic studies in *E. coli* K12 have implicated several genes that play a role in the rate of formation of both dormant and persister cells. Many of these genes encode toxin-antitoxin (TA) modules [7,8,11]. One example is *hipA* (high persistence). One allele of this gene (*hipA7*) causes a 100 to 1000-fold increase in persister levels [12], and over-expression of *hipA* leads to growth arrest and a persistence phenotype [13]. Several other loci have also been associated. Maisonneuve et al. [11] recently showed that overexpression of any one of five toxins from mRNase TA pairs resulted in higher fractions of persisters for both ciprofloxacin and ampicillin. In addition, by serially deleting up to ten TA loci, the authors showed that decreasing the number of TA loci decreased the fraction of persisters. Deleting ten TA loci decreased the persister fraction by 100-fold, from approximately 1% to 0.01% after five hours of antibiotic treatment, and this decrease occurred for both ciprofloxacin and ampicillin. The authors proposed a model in which mRNase toxins inhibit global translation, cells become dormant, and thus persist. These data suggest that in *E. coli* K12, a substantial fraction of persisters arise through mechanisms involving mRNase TA loci (deleting all ten loci results in a 99% reduction in persister frequency; deleting any one locus results in only an approximately 10% reduction in persister frequency). It is unknown whether similar mechanisms are important in other bacteria.

Other than *E. coli* K12, the majority of persister studies have focused on three bacterial taxa: *Mycobacterium tuberculosis, Pseudomonas aeruginosa*, and *Staphylococcus aureus*. *M. tuberculosis* is known for its recalcitrance to antibiotic treatment [14-16], and genetic studies have shown that toxin overexpression exhibits drug-specific effects: toxins that increase persistence in one antibiotic do not necessarily increase persistence in other antibiotics [15]. This contrasts with results in *E. coli* K12 outlined above, in which persistence is generally characterized by multidrug tolerance [9,11]. In clinical settings, *P. aeruginosa* mutants that produce increased persister fractions (up to 100-fold above wildtype) have been isolated [4]; however, the genetic mechanisms causing increased persister fractions are not well understood. Finally, in *S. aureus*, although some research on the influence of metabolism on persister formation [17], genetic studies are lacking.

Most studies on persister formation have focused on strains harboring mutations that increase or decrease persister frequency. However, one recent study [18] tested how persister formation differs among strains of bacteria. In this study, mammalian commensal and pathogenic *E. coli* isolates were found to exhibit substantial variation in the fraction of persisters that are present in exponentially growing populations of cells. In addition, it was found that the fraction of persisters that survived treatment in one antibiotic was uncorrelated with the fraction surviving in a second antibiotic. However, without a quantitative model of persistence, this result cannot unambiguously exclude other explanations, such as differences in the death rates of cells between isolates.

Here, using a collection of environmental isolates of *E. coli*, we examine variation in the frequency of persister cells in naturally occurring strains. In order to consistently measure persister fractions, we use a mathematical model to derive quantitative and reliable estimates of the fraction of persisters in each population. Our quantitative set of data corroborates the results of the previous study on commensal and pathogenic *E. coli* isolates [18], showing that there is substantial variation in the fraction of persister cells among environmental isolates of *E. coli*. In addition, we show that the fraction of cells that survive drug treatment in one drug is uncorrelated with the fraction surviving in a second drug. Importantly, we show that this lack of correlation extends to drugs have nearly identical modes of action. Finally, by using combinations of antibiotics, we provide evidence that for any single strain, there may be a subset of persister cells that are recalcitrant to treatment with any antibiotic. In particular, although treatment with different antibiotics results in different fractions of persister cells for any one strain, treating that strain with combinations of antibiotics frequently selects for the subset of cells that persists in the face of the most harmful antibiotic.

Together, these data imply that the ability of cells to persist in the face of antibiotic treatment depends on the specific mechanism by which the persister phenotype is generated, and the precise manner in which the antibiotic acts: cells that persist in one antibiotic may not persist in a second antibiotic, even if that antibiotic has a very similar mode of action. These data contrast strongly with data from experimental studies on lab strains of *E. coli*, which have generally shown that when mutants exhibit higher levels of persistence in one antibiotic, they also exhibit higher levels of persistence in other antibiotics (multidrug tolerance) [6,7,11,13,19-22]. However, there do appear to be a subset of cells that persist after treatment with multiple antibiotics, as evidenced by using combination treatments. Finally, the data here suggest that the parameter that has the largest influence on the fraction of persisters exhibited by any strain is the rate of switching from a normal cellular phenotype to a persister state; in contrast, the rate of switching from persister to normal cell has a much smaller influence.

## Results

### Consistent quantification of persister fractions

A critical issue when studying bacterial persistence is the precise definition of the persister fraction. Previous studies have defined persister cells as the surviving fraction after antibiotic exposure for an arbitrary amount of time, ranging from hours [4,8,10,11,19,23-25] up to several days [15]. In addition, these fractions have been assessed at different growth states: mid-exponential [8,10,11,19,25], late exponential [24] and in rare cases, stationary phase [4,24,25]. Most often, these studies are performed in liquid cultures of rich media. However, some studies have assayed persisters on agar [6,12,13], by plating samples of logarithmically growing cultures on LB agar with ampicillin, incubating overnight, spraying the plates with penicillinase, and again incubating for 24 hours to count the number of surviving cells. These different methods tremendously complicate comparisons across studies.

To quantify the fraction of persisters in a consistent manner, we use a model motivated by observations of persister cell dynamics first reported by Balaban et al. [6], who observed two types of persister cells, which they proposed arose through two different mechanisms. Type I persisters occurred through unspecified events that occur during stationary phase, and remained fully dormant until switching to a normal growth state. These have been associated with a specific genotype, the *hipA7* allele. Type II persisters arise through an infrequent stochastic switch to a slow-growth state, and remain so until switching to a normal growth state. These were associated with a mutation at a second locus, *hipQ*. A similar model of persister formation has been proposed by Wiuff et al. [23].

Here we apply this simple two-state model assuming simplified type II persister dynamics. In this model, cells exist in two states, normal and persister. During antibiotic treatment, normal cells die at a rate μ and switch to a persister state at rate α. Persister cells do not die or grow, and switch to a normal state at rate β (see Additional file 1). The advantage of using a this model is that the parameters that we infer, such as the fraction of persister cells, do not depend on experimental idiosyncrasies, for example, the time at which cell numbers are measured. It has been difficult to compare the results of many previous experiments on persisters for this reason.

### Persister fractions differ between environmental isolates

We selected 11 *E. coli* isolates from a collection of more than 450 environmental isolates sampled over a period of 12 months from two sites approximately 2m apart near a watershed of Lake Superior (46°42'04'N, and 92°12'26'W) [26]. Despite the nearly identical geographical provenance of these isolates, partial genomic sequencing of a subset of these 450 strains has shown that while all are *Escherichia* species, they encompass a genetic diversity greater than the standard panel of *E. coli* strain diversity, the ECOR collection. This initial genomic data show that isolates from this location are spread across the *E. coli* phylogeny, with members in clades A, B1, B2, D, E, F, and C-V [27] (Bertels et al., in prep). Although the strains in this collection harbor considerable genetic diversity, for the most part, they are not pathogenic, typing negatively for most common virulence loci (M. Sadowsky, personal communication).

We selected the subset of 11 environmental isolates on the basis of their differential levels of survival in ampicillin after 24 hours of treatment (using CFU counts; see Methods). In doing so, we aimed to find strains that differed to the greatest extent in the fraction of persisters that were formed in ampicillin, such that we would have the greatest power to discern whether these differences were paralleled in other antibiotics. In addition to these isolates, we used the standard laboratory strain *E. coli* K12 MG1655, for a total of 12 strains in which we quantified persister fractions.

For each of these strains, we first determined the MIC for ampicillin (see Methods), and found that the MICs for these strains differed by less than two-fold (Additional file 2: Table S1). This suggested that the differences in survival did not arise simply from differences in growth and killing dynamics, and may instead have resulted from differences in persister formation.

**Figure 1:**
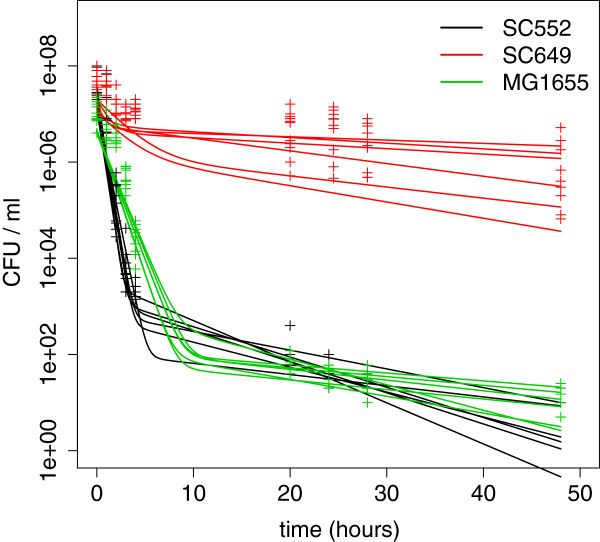
**Environmental isolates exhibit substantial variation in persister fractions after treatment with 100 ug ampicillin.** The kill curves are characterized by biphasic behavior, implying that there are at least two distinct populations of cells with differing death rates. The plot shows the killing data of six replicate cultures for three strains (SC552, SC649 and MG1655); the lines indicate the best-fit models for each replicate.

We then quantified, for each strain, survival curves over 48 hours during treatment with 100 mg/ml of ampicillin (Figure 1). In the vast majority of cases, the curves that we observed were clearly not characterized by a single exponential decrease, as would be expected if all individuals in the population had equal susceptibility to the antibiotic. This suggested that at least two distinct populations of cells were present. We denote these subpopulations as normal and persister cells. We used these survival curves in conjunction with a mathematical model of persistence to quantify the persister fraction for each strain. In this model we fit four independent parameters (see Additional file 1) to infer the rate of death of normal cells, the rates of switching between normal and persister states, and the fraction of persisters. For each strain, we used at least five biological replicates for model fitting.

**Figure 2:**
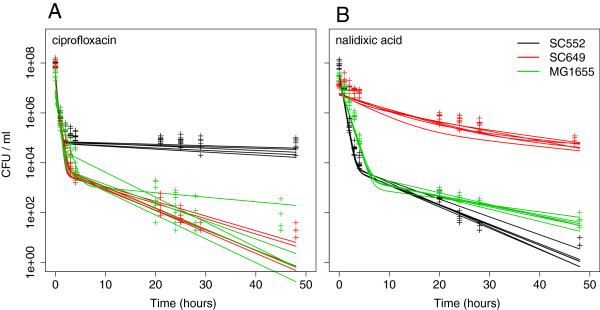
**Environmental isolates exhibit different fractions of persisters after treatment with ciprofloxacin or nalidixic acid.** The plots show six replicates for each of the three strains shown in Figure 1. **A**: Killing dynamics during 48 hours of treatment with ciprofloxacin. Biphasic dynamics, similar to those observed in Figure 1, are observed. **B**: Killing dynamics during 48 hours of treatment with nalidixic acid. There are large differences in persister fractions between the two antibiotics, with strain SC649 exhibiting a low fraction of persisters in ciprofloxacin, but a high fraction in nalidixic acid.

Using this method, we found that the fraction of persisters differed significantly between strains, from less than 0.001% to more than 10% (Figures 1 and 2; Additional file 3: Table S2), a range of over four orders of magnitude.

### Persister fractions in different antibiotics are uncorrelated

To infer persister fractions, we also measured kill curves for each strain in two additional antibiotics, ciprofloxacin and nalidixic acid, both belonging to the quinolone class of antibiotics [28]. By selecting two antibiotics in the same class, we aimed to test whether persister fractions were similar and consistent for drugs with comparable modes of action. We first measured the MICs of these 12 strains in both antibiotics, and found that the MIC values showed little variation (differing by 2.5-fold and 3.5-fold for ciprofloxacin and nalidixic acid, respectively; Additional file 2: Table S1). We used the same method outlined above to quantify the persister fractions in these antibiotics. We again found substantial variation in the persister fractions, ranging from 0.001% to 0.15% in ciprofloxacin, and from less than 0.001% to more than 1% in nalidixic acid (Additional file 3: Tables S2).

Our hypothesis is that for each strain, persisters are generated through a single general mechanism, such as cell dormancy, and that this mechanism confers a multi-drug tolerance. If this is true, then strains should exhibit characteristic persister fractions: we expect that for some isolates this subset of cells will be large, and thus these isolates will have high fractions of persisters across all antibiotics, while for other isolates, this subset of cells will be small, resulting in a small fraction of persisters across all antibiotics. This pattern has been shown previously for the *hipA7* mutant of *E. coli* K12, after *relE* overexpression in K12, or after deletion of TA-pairs [11,29,30]. In all of these cases, these genetic changes caused a general increase in the fraction of persisters across several classes of antibiotics.

**Figure 3:**
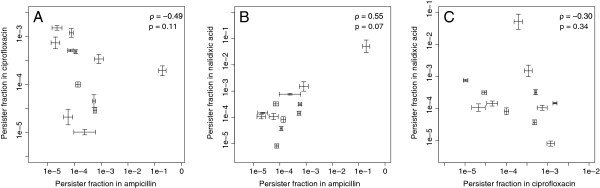
**No correlation is observed between persister fractions in different antibiotics.** We found that although the calculated persister fractions are repeatable, there is no consistent correlation between the fractions of persisters in any two antibiotics. The plots show the correlations in persister fractions. **A**: ampicillin and ciprofloxacin; **B**: ampicillin and nalidixic acid; and **C**: ciprofloxacin and nalidixic acid. Only one strain exhibits a very high fraction of persisters in two antibiotics; however, these antibiotics are ciprofloxacin and ampicillin, members of two different classes. The error bars indicate standard errors for the biological replicates. The values of Spearman’s rho and the corresponding p-value are shown in each plot.

We tested this hypothesis by looking for positive correlations in the fraction of persisters in the three antibiotics (ampicillin, ciprofloxacin, and nalidixic acid). However, despite the considerable variation in the persister fractions found among isolates (Figure 2), no consistent positive correlations were found (rho = -0.49, p = 0.46, N = 12 for ampicillin versus ciprofloxacin, rho = 0.55, p = 0.07, N = 12 for ampicillin versus nalidixic acid, rho = −0.30, p = 0.34, N = 12 for ciprofloxacin versus nalidixic acid, Spearman correlation; Figure 3). Importantly, we found no positive correlation between the persister fractions in ciprofloxacin and nalidixic acid, although these two antibiotics have very similar mechanisms of action, with both targeting the DNA gyrase subunits *gyrA* and *gyrB* and the topoisomerase IV subunits *parC* and *parE*[31,32]. It is unlikely that this result is due to an inability to accurately measure the persister fractions, as independent measurements yielded highly consistent values (Figures 1 and 2). Thus, this result suggests that different types of persister cells exist within populations, some of which are persistent to one antibiotic, while others are persistent to other antibiotics. In addition, this shows that *E. coli* persister cells are not necessarily characterized by multidrug tolerance. Although this contrasts with previous observations for mutants of *E. coli* K12, it is in concordance with observations in *M. tuberculosis*[15].

### Evidence that a subset of persister cells is multidrug tolerant

**Figure 4:**
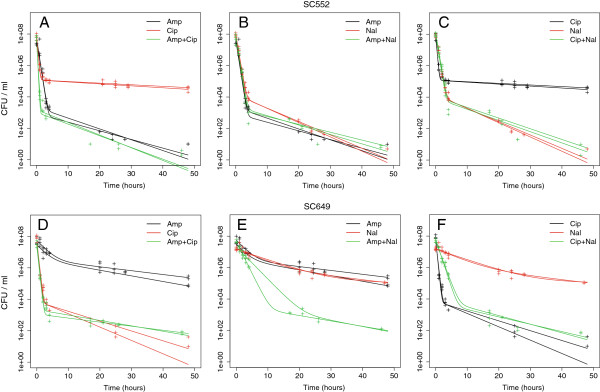
**Kill curves in combinations of antibiotics are biphasic and vary between treatments.** We used combinations of antibiotics to examine the dynamics of cell killing. These dynamics are similar to those observed in single antibiotics. **A–C**: Killing dynamics of all replicate cultures upon treatment of strains SC552 with all pairwise combinations of the three antibiotics. **D-F**: Killing dynamics of strain SC649.

We selected two strains on the basis of the persister fractions that they exhibited in single antibiotics, requiring that the strains exhibit a high level of persistence in at least one antibiotic. For these two strains we re-measured the persister fractions in single antibiotics, as well as in all pairwise combinations of the three antibiotics. We found that the killing dynamics were qualitatively similar to those when using a single antibiotic: all kill curves exhibited biphasic behavior, indicating that at least two subpopulations of cells were present (Figure 4).

The precise dynamics of this killing in combinations of antibiotics may yield additional insight into how persisters are formed. We briefly outline three general possibilities. (1) No cells persist when a population is simultaneously treated with antibiotics. This implies that the mechanisms underlying persistence to the two antibiotics are exclusive, and cannot occur within the same cell. (2) The fraction of persistent cells under the combination of antibiotics is approximately multiplicative relative to the fraction in the two single antibiotics. Although this observation would be consistent with several explanations, the simplest is that the mechanisms of persister formation are independently induced, and occur randomly within the same cell. (3) The fraction of persistent cells under a combination of antibiotics is similar to the fraction observed under treatment with the more lethal antibiotic. Again, although several explanations would be consistent with this, the simplest is that cells that are persistent to the more lethal antibiotic are also persistent to the second. We refer to these three hypotheses as exclusive, independent, and coincident, respectively.

**Figure 5:**
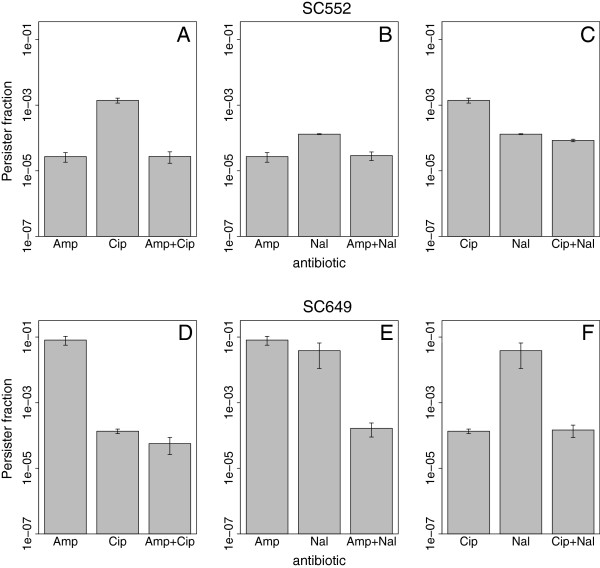
**A subset of persister cells is multidrug tolerant.** The persister fractions estimated from the killing dynamics are shown for single or combinations of antibiotics. **A**: strain SC552; **B**: SC649. For both strains, there is a subset of persisters that appear to be resistant to both antibiotics.

We found that for these two strains, there were no cases in which persister fractions were exclusive. Instead, the persister populations were largely coincident, with the fraction of cells in combinations of antibiotics being similar to the fraction observed in the more lethal antibiotic (Figures 4 and 5). This is consistent with this subset of cells being multidrug tolerant. Thus, although not all persisters are multi-drug tolerant, there appears to be a subset that is.

### Toxin-antitoxin pairs are frequently gained and lost in *E. coli* isolates

**Figure 6:**
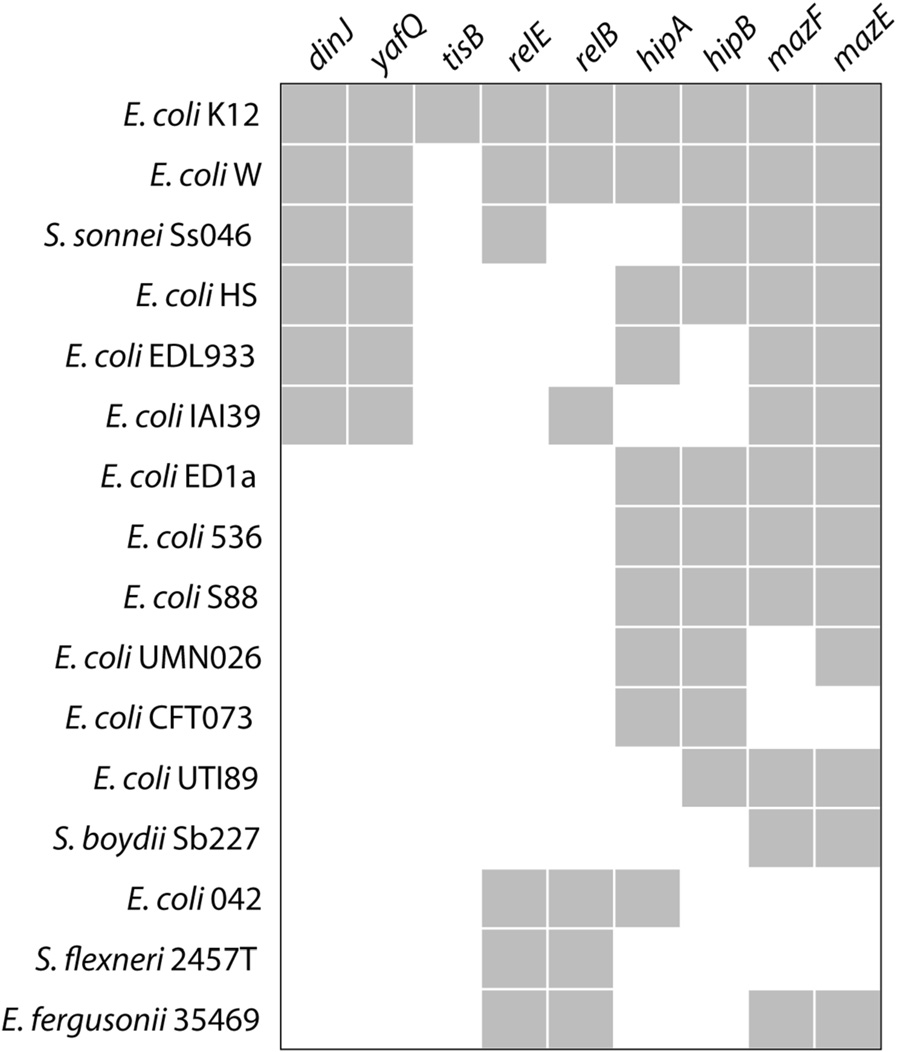
**Known persister loci are rapidly gained and/or lost within the *E. coli *clade.** Grey boxes indicate the presence of the orthologue in the indicated genome; white indicates absence. The data suggests that toxin – antitoxin loci undergo rapid loss and/or gain within the *E. coli* clade. Orthologue presence – absence of toxin-antitoxin loci is based on a bidirectional best-hit analyses [33] for 14 *E. coli* and *Shigella* taxa and *E. fergusonii*.

One possible explanation for the differences in persister formation of environmental isolates is that the activation of different toxin-antitoxin pairs results in different antibiotic susceptibilities. To further examine this hypothesis, we looked at the presence of TA loci that are known to affect persister formation in 15 *E. coli* and *Shigella* taxa, as well as in *Escherichia fergusonii*. We found significant variation in the presence of TA modules across different *E. coli* isolates (Figure 6), suggesting that these loci are lost (and/or gained) over relatively short time scales in this clade. Such changes in the number or types of TA pairs are likely to affect the production of persister cells, as has been shown experimentally [11].

### The rate of switching from normal to persister state is the primary determinant of persister fractions

**Figure 7:**
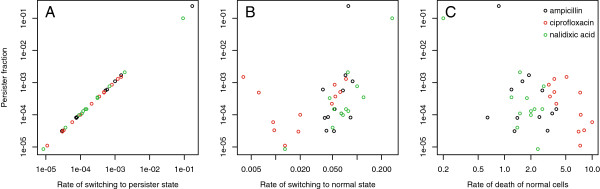
**The primary determinant of the persister fraction is the rate of switching to the persister state. A**: The rate of switching from the normal cellular state to the persister state is strongly correlated with the fraction of persisters in the population. **B**: There is little to no correlation between the rate of switching from the persister state to the normal state and the fraction of persisters. **C**: No correlation exists between the rate of death of normal cells and the persister fraction.

In the analyses above, we have used information from cell-killing dynamics to infer the proportion of persister cells that were present at the start of antibiotic killing. These persisters are formed during exponential growth, and the fraction that is present is determined largely by two independent parameters, the rates of switching to and from the persister cell state. To gain additional insight into the mechanistic underpinnings of persister formation, we examined the relationship between the persister fraction and these two parameters. We find strong evidence that the primary determinant of the persister fraction is the rate at which persister cells are formed from normal cells: these two variables are strongly correlated across both strains and antibiotics (Figure 7). In contrast, the rate of switching from persister to normal cell has little to no relationship with the persister fraction.

## Discussion

In generating antibiotic kill curves from CFU data, we have shown that these curves differ substantially between environmental isolates of *E. coli* for single antibiotics. In addition, we found that the shape of these curves differs between different antibiotics. Using a quantitative theoretical model of the relationship between the shape of these curves and persister formation, we have been able to show that these results imply that persister fractions differ between environmental isolates and between antibiotics. Our results imply that there are no *E. coli* strains that have generally high or low levels of persisters; instead, there are different types of persister cells within populations, and each type may be more or less persistent to different antibiotics. Importantly, the variation in persister fractions exists even for antibiotics with nearly identical modes of action (ciprofloxacin and nalidixic acid). Mechanistically, this suggests that persistence through cell dormancy is not a single, general phenomenon. Instead, there may be distinct physiological states of dormancy, each of which is differently susceptible to a particular antibiotic. The idea that there are different types of persister cells that arise from a variety of mechanisms has also been proposed in a recently published study [34].

We note that one complicating factor in this interpretation is that these different persister populations may have different propensities to form colonies, and that this might explain some of the differences in the shapes of the kill curves that we observed. However, given the range of persister fractions that we observed (over four orders of magnitude), we do not think that this mechanism can fully explain the patterns that we find.

It is also possible that although the isolates that we studied have similar MIC values, they differ in their pharmacodynamics [35]. However, the persister fraction should largely be independent of the pharmacodynamic behavior; thus this is unlikely to account for the differences that we observe between isolates [34].

Evidence of two different types of persister cells has been shown previously by Balaban et al. [6], and genotypic changes at different loci were associated with each phenotype. Similarly, genetic differences between different *E. coli* isolates, such as the presence or absence of TA various modules, may affect the production of persister cells (Figure 6).

Gefen et al. [36] suggested that large differences in the measurement of persister fractions might arise because antibiotic exposure begins at different stages of exponential growth (before or after 1.5 hours of growth). However, by growing the cells for four hours, we hope to have minimized such effects, and propose that the large differences we find in persister fractions are not due to differences in growth stage, but to fundamental differences in the mechanisms of persister production.

We note that the set of environment isolates that we have used are not known to be pathogenic, suggesting that many of them have had less exposure to antibiotics and the concomitant selection for resistant or persister phenotypes that arises from such exposure. In previous studies of clinical isolates, selection has been shown to result in rapid changes in the frequency of genotypes differing in their ability to form persisters [4]). However, our results suggest that even in the absence of recent bouts of antibiotic-mediated selection, we find that persister fractions differ considerably among different genotypes, suggesting that variation in persister-forming ability is harbored naturally in populations.

Previous studies have indirectly implied that mechanisms of persister formation may differ between strains for different antibiotics. Keren et al. [7] showed that one strain of *E. coli* K12 (AT984 *dapA zde-264::Tn10*) exhibited a higher fraction of persisters in ofloxacin compared to ampicillin, whereas Spoering et al. [24] showed the reverse: *E. coli* K12 wildtype exhibits a lower fraction of persisters in ofloxacin than ampicillin. For both studies, the drugs were used at identical concentrations (5 ug/ml and 100 ug/ml, respectively). Again, this result suggests that even for *E. coli* K12, closely related mutants do not necessarily produce large or small persister fractions, but these fractions depend specifically on the type of antibiotic and strain used.

To our knowledge, the effect of pairwise combinations of antibiotics has not been investigated with respect to bacterial persistence. We found that the killing dynamics under combinations was qualitatively similar to that observed under a single antibiotic, with biphasic kill curves. Furthermore, the observation of co-incident persister fractions provide evidence that there is a small number of persister cells that exhibit multidrug resistance, and are thus persistent to all combinations of antibiotics (Figure 5). However, the majority of persister cells do not exhibit multidrug-resistance.

## Conclusions

The results of our study clearly show that the fraction of persisters within an isogenic culture is highly dependent on the antimicrobial compound and the bacterial strain. Importantly, differences in persister fractions exist even for antibiotics of the same class. This contrasts markedly with the majority of laboratory studies of *E. coli* K12, which have generally found that persister phenotypes are characterized by multi drug tolerance. These results complicate the search for persister mechanisms, since even within the same strain different types of persister cells exist, with none clearly dominating.

## Methods

### Strains

The *E. coli* natural isolates used in this study were selected from a collection of 456 *E. coli* sampled from a watershed of Lake Superior, Minnesota, USA (46°42'04'N, and 92°12'26'W [26]; Additional file 2: Table S1). For this study, all strains were treated with ampicillin (100 μg/ml) for 24 h, and 11 strains that showed marked differences in survival (as measured by colony counts) were selected.

### Media

M9 salts supplemented with 0.2% glucose was used as a growth medium in all experiments.

### Determination of minimum inhibitory concentrations (MICs)

Single colonies were used to inoculate 200 μl of M9 salts supplemented with 0.2% glucose in 96-well plates. The plates were incubated overnight at 37°C with shaking at 400 rpm. These overnight cultures were diluted 1:100 into fresh medium and incubated for 2 h at 37°C with shaking at 400 rpm to ensure logarithmic growth. Approximately 5 × 10^7^ cells were then used to inoculate 150 μl of M9 containing different concentrations of antibiotics and all wells were covered with 50 μl mineral oil to avoid evaporation. Growth was assessed by measuring the optical density (OD) at a wavelength of 600 nm over 20 hours using a plate-reader system from BioTek. The lowest concentration of antibiotic that did not exceed an OD of 0.01was taken to be the MIC of that antibiotic for a particular strain.

### Antibiotic kill curves

Single colonies were used to inoculate 200 μl M9 minimal medium supplemented with 0.2% Glucose. The plates were incubated overnight at 37°C with shaking at 400 rpm. The overnight culture was diluted 1:100 into 1.5 ml fresh medium in a 24-well plate and incubated at 37°C with shaking at 250 rpm for 4 h to ensure logarithmic growth of the cultures. After 4 h of incubation, antibiotics were added at the following concentrations: 100 μg/ml ampicillin, 0.1 μg/ml ciprofloxacin and 150 μg/ml nalidixic acid. In preliminary experiments using kanamycin, we found that regrowth frequently occurred, despite a secondary spiking of the culture with kanamycin. This suggested that resistance often arose [37], and we did not pursue this drug further. After the addition of the antibiotics, hourly samples were taken for the first 4 h, serially diluted in phosphate buffered saline (PBS) and spot-plated in 5 μl drops onto LB agar plates to determine colony-forming units (CFU). Additional samples were taken at 20, 24, 28 and 48 hours (with slight variations) after addition of the antibiotic and 100 μl–500 μl were plated to LB agar plates, depending on the counts of previous time points. All assays were performed using 6 replicates and all plates were counted at least twice on different days (after 24 and 48 hours) to ensure the detection of late appearing colonies [38]. Surviving colonies were tested for resistance to the respective drug they were treated with and replicates with resistant cells were excluded from the analysis. For the three antibiotics in which we present data on here (nalidixic acid, ampicillin, and ciprofloxacin), resistance was rarely observed, and only with ciprofloxacin and nalidixic acid.

For a subset of cases, we repeated the kill curve measurements using colonies that survived 48 hours of antibiotic treatment. In all cases, we observed dynamics similar to those observed for the original culture (data not shown), showing that these cells are likely to differ only in a phenotypic, and not genotypic, manner. In addition, we spiked the cultures with additional antibiotic after 24 hours, and found that this had no significant effect on the killing dynamics, showing that the dynamics we observe are not due to degradation of the antibiotic. Assays using combinations of antibiotics were performed similarly to those outlined above, with the antibiotics added at the same concentrations as they were in the single-drug assays. In this case, the experiments were performed in duplicate.

### Quantification of persister fractions

The fraction of persisters, death rates and switching rates between persister and normal states were calculated using a model motivated by Balaban et al. [6]. In this model, cells switch between two states, normal and persister. The equations describing the dynamics of this switching is detailed in the Additional file 1, together with the exact solutions of these coupled differential equations.

We used maximum likelihood to fit the CFU count data, under the assumption that the error in the CFU counts results primarily from Poisson sampling, using the likelihood function:

$$\begin{array}{l} \Lambda \left(c_{1},c_{2},\lambda_{1},\lambda_{2}|x\right)=\prod_{t=0}^{t=T}\frac{{\left(\frac{N\left(t|c_{1},c_{2},\lambda_{1},\lambda_{2}\right)}{\mathrm{\delta t}}\right)}^{x_{t}}}{x_{t}!}\\ \;\times \exp(\frac{N(t|c_{1},c_{2},\lambda_{1},\lambda_{2})}{\delta_{t}}) \end{array}$$

in which *x*_*t*_ is the number of CFUs observed at time point *t*, *δ*_*t*_ is the dilution at time point *t*, and N(t) is the number of cells predicted by the model (see Additional file 4). The values that these parameters can take are restricted, as outlined in the Additional file 1.

Likelihood maximization was done using *optim()* in the R statistical framework [39]. Likelihood convergence was checked by using ten separate starting values for the parameters and three optimization algorithms, Nelder-Mead, SANN, and BFGS. The values of the a, b, m, and F_0_ (the initial fraction of persisters) were determined independently for each replicate, and we calculated confidence intervals assuming normally distributed error. Because the values of a, b, and m cannot be uniquely fit (see Additional file 1), we calculated them using the median value of F_0_; in most cases, the uncertainty in F_0_ is very low, with most minimum and maximum values of F_0_ ranging between 0.99 and 1. Thus, this approximation has little effect on our data.

All other statistical analyses were performed using R [39].

## Authors’ contributions

NH participated in the experimental design, collected all experimental data, performed the data analysis, and drafted the manuscript. EvN participated in the experimental design, performed the analytical derivations, and edited the manuscript. OKS conceived and designed the project, performed the computational and bioinformatic analyses, and drafted the manuscript. All authors read and approved the final manuscript.

## Supplementary Material

Additional file 1 Appendix. Click here for file

Additional file 2: Table S1 Minimum inhibitory antibiotic concentrations for each strain. The MICs ranged between 15-22.5 μg/ml for ampicillin, between 0.008-0.030 μg/ml for ciprofloxacin and 3-7.5 μg/ml for nalidixic acid. This variation in MICs was considerably smaller than the variation in persister fractions exhibited by the selected strains and moreover, the fraction of persisters and their corresponding MICs showed no correlation, suggesting that the variation in MICs does not account for the one observed in the level of persister cells. No resistance to the three used antibiotics was evident for any of the examined. Click here for file

Additional file 3: Table S2 Estimated death rates and switching rates for all strains in the three antibiotics (ampicillin, ciprofloxacin, and nalidixic acid). The parameters are explained in the Additional file 1. Electronic supplementary material. Click here for file

Additional file 4 **R code for maximum likelihood fitting.** The R code used to perform the fits of the data is provided. Click here for file
