# Supplementary material for: Quantitative analysis of persister fractions suggests different mechanisms of formation among environmental isolates of E. coli
Source: BMC Microbiol. 2013 Feb 4;13:25. doi: 10.1186/1471-2180-13-25 (PMC3682893; doi:10.1186/1471-2180-13-25)
Supplement: Additional file 1 — Appendix. [file 1471-2180-13-25-S1.pdf]

Appendix to:
Quantitative analysis of persister fractions suggests different mechanisms of formation among environmental isolates of E. coli

Niels Hofsteenge Erik van Nimwegen
Olin Silander
Core Program Computational and Systems Biology, Biozentrum, University of Basel,
Klingelbergstrasse 50-70, 4056 Basel, Switzerland

Below we introduce a model of the dynamics of an isogenic cell culture under treatment with a lethal dose of antibiotic. Following Balaban et al. [1], we assume cells stochastically switch between a normal state, which is susceptible to the antibiotic, and a persister state, which is not susceptible to the antibiotic.

Differential equation model

We assume that at the start of the experiment there are n_0 normal cells and p_0 persisters. We further assume that, during the antibiotic treatment, normal cells are killed at an effective rate μ (which is the difference of their death and proliferation rates), whereas persisters are assumed to neither die nor proliferate (i.e. an effective growth rate of zero). Finally, we assume normal cells switch to a persister state at rate a, and persisters revert to normal cells at rate b. This leads to the following set of differential equations

$$\frac{dn}{dt} = -(a+\mu)n + bp, \tag{1}$$

for the number of normal cells n, and

$$\frac{dp}{dt} = an - bp \tag{2}$$

for the number of persisters p.

Since we cannot directly distinguish persisters from normal cells, our measurements only give access to the total number of cells $T = n + p$ as a function of time. Note that

$$\frac{dT}{dt} = \frac{dn}{dt} + \frac{dp}{dt} = -\mu n \tag{3}$$

If we define $f = n/T$ to be the fraction of normal cells, we find

$$\frac{dT}{dt} = -\mu f T, \tag{4}$$

which has the general solution

$$T(t) = T_0 \exp\left(-\mu \int_0^t f(\tau)d\tau\right) = T_0 e^{-\mu\langle f\rangle t}, \tag{5}$$

with $\langle f \rangle$ the time averaged fraction of normal cells from time 0 to time t. We derive this equation (5) because it gives an intuitive insight into the dynamics of the total number of cells. Initially most cells are normal, i.e. $\langle f \rangle$ is close to 1, and $T(t)$ drops exponentially at a rate close to μ. As many of the normal cells die, the fraction of persisters starts rising, $\langle f \rangle$ becomes smaller, and the decay rate starts dropping. Eventually a steady-state fraction f_* of normal cells is reached, and $\langle f \rangle$ will approach this steady-state fraction f_*. From that point onward, $T(t)$ will again follow simple exponential decay, but now with rate μf_*. That is, the decay rate at late times is simply the product of the limit fraction of normal cells f_* and the death rate μ of normal cells.

Persister fraction

To calculate the limiting persister fraction f_* and the dynamics of f we can write a differential equation for f. We have

$$\frac{df}{dt} = \frac{d(n/T)}{dt} = \frac{1}{T}\frac{dn}{dt} - \frac{n}{T^2}\frac{dT}{dt}. \tag{6}$$

Using the equations for dn/dt and dT/dt already derived above we find

$$\frac{df}{dt} = b - (a + b + \mu)f + \mu f^2. \tag{7}$$

Note that this equation does not depend on T. From it we can easily calculate the fraction f_* at late times, i.e. by solving

$$\mu f^2 - (a + b + \mu)f + b = 0. \tag{8}$$

To simplify this equation, we note that we can divide out an overall factor μ. By defining relative rates $\alpha = a/\mu$, $\beta = b/\mu$ and $\gamma = (\alpha + \beta + 1)$, we obtain

$$f^2 - \gamma f + \beta = 0. \tag{9}$$

The solution (wich lies in $0 \leq f_* \leq 1$) is

$$f_* = \frac{\gamma}{2}\left(1 - \sqrt{1 - \frac{4\beta}{\gamma^2}}\right). \tag{10}$$

To develop some intuition for this expression, we note that $\gamma > 1$ and that β must be a small number (i.e. $b \ll \mu$) because the death rate is much larger than the rate at which persisters switch back to normal cells. If this were not the case, we wouldn't notice there were persisters. We thus expand to first order in β and find

$$f_* \approx \frac{\beta}{1 + \alpha + \beta} = \frac{b}{a + b + \mu}. \tag{11}$$

That is, to first order, the fraction of normal cells is given by the relative rate at which they are produced (b) from persisters, and the total rate ($a + \mu$) at which they disappear.

Dynamics at all times

The solution of the differential equation for $T(t)$ takes the following general form

$$T(t) = T_0 \left[c_1 e^{-\lambda_1 \mu t} + c_2 e^{-\lambda_2 \mu t} \right], \tag{12}$$

where the exponents are given by

$$\lambda_1 = f_* = \frac{\gamma}{2}\left(1 - \sqrt{1 - \frac{4\beta}{\gamma^2}}\right), \tag{13}$$

and

$$\lambda_2 = \frac{\gamma}{2}\left(1 + \sqrt{1 - \frac{4\beta}{\gamma^2}}\right). \tag{14}$$

Note that $0 \leq \lambda_1 \leq 1$ and that $\lambda_2 \geq 1$.

The prefactors can also be expressed in terms of the exponents and the initial fraction of normal cells f_0:

$$c_1 = \frac{\lambda_2 - f_0}{\lambda_2 - \lambda_1}, \tag{15}$$

and

$$c_2 = \frac{f_0 - \lambda_1}{\lambda_2 - \lambda_1}. \tag{16}$$

Note that, per definition, $c_1 + c_2 = 1$, i.e. these are not two independent parameters. Note also that because $\lambda_2 > \lambda_1$ it is guaranteed that $c_1 > 0$, i.e. in the limit of long time the number of cells is positive, as it should be.

Fitting

The most straight-forward approach to fitting this model is to introduce the five parameters T_0, μ, α, β, and f_0, and then search for the maximum likelihood solution, i.e. where the observed data are maximally likely given the predicted dynamics $T(t)$. However, this set of 5 parameters is *redundant* in that different settings of these parameters can lead to the *same* predicted dynamics $T(t)$.

Thus, instead of fitting these 5 parameters directly, we instead fit a general mixture of two exponentials, i.e. we introduce the general solution $T(t) = \tilde{c}_1 e^{-\tilde{\lambda}_1 t} + \tilde{c}_2 e^{-\tilde{\lambda}_2 t}$. However, not every combination of parameters $(\tilde{c}_1, \tilde{c}_2, \tilde{\lambda}_1, \tilde{\lambda}_2)$ can be realized by our model. Specifically, allowing any combination $\alpha \geq 0$, $\beta \geq 0$, and $\mu \geq 0$, $0 \leq f_0 \leq 1$, and $T_0 \geq 0$, we find that

- Any $\tilde{c}_1 \geq 0$ can be realized.
- Any $\tilde{c}_2 \geq -\tilde{c}_1$ can be realized. Note that $\tilde{c}_1 + \tilde{c}_2 = T_0$, and T_0 has to be non-negative. It is thus more natural to pick $\tilde{c}_1$ and T_0 freely, and then set $\tilde{c}_2 = T_0 - \tilde{c}_1$.
- Any value $\tilde{\lambda}_1 \geq 0$ for the 'slow' exponent (i.e. $\tilde{\lambda}_1$ is per definition smaller than $\tilde{\lambda}_2$) can be realized.

- Given that we freely pick $(T_0, \tilde{c}_1, \tilde{\lambda}_1)$, the value of $\tilde{\lambda}_2$ is constrained as follows. If we write

$$\tilde{\lambda}_2 = \tilde{\lambda}_1(1+\Delta), \tag{17}$$

then Δ has to lie in the interval $[0, T_0/\tilde{c}_1]$.

In order to ensure that the parameters $(\tilde{c}_1, \tilde{c}_2, \tilde{\lambda}_1, \tilde{\lambda}_2)$ satisfy these constraints we use the following parametrization. Let (x, y, z, w) be an arbitrary vector of 4 real numbers. Then write

- $\tilde{c}_1 = e^{y+x}$.
- $\tilde{c}_2 = e^{y}(1-e^{x})$.
- $\tilde{\lambda}_1 = e^{z}$.
- $\tilde{\lambda}_2 = e^{z}\left(1 + \frac{e^{-x+w}}{1+e^{w}}\right)$.

Determining the parameters from the general fit

Once we have determined maximum likelihood values of (x, y, z, w), and through them the values of $(\tilde{c}_1, \tilde{c}_2, \tilde{\lambda}_1, \tilde{\lambda}_2)$, we want to determine the values of the parameters (a, b, μ, T_0, f_0) that correspond to these parameters. Of course, we generally cannot determine all 5 parameters from the 4 fitted constants. What we will thus do, is determine α, β, μ, and T_0 in terms of the fitted parameters and f_0. We will see below that, for realistic parameters, f_0 may be tightly constrained in small window near $f_0 \approx 1$. We have for the parameters in terms of the fit:

- $$T_0 = \tilde{c}_1 + \tilde{c}_2 = e^{y}$$
  .
- $$\mu = \frac{\tilde{\lambda}_1}{f_0}(1+\Delta-\rho\Delta),$$
  where $\rho = \tilde{c}_1/T_0 = e^{-x}$ and $\Delta = (\tilde{\lambda}_2 - \tilde{\lambda}_1)/\tilde{\lambda}_1 = e^{-x+w}/(1+e^{w})$.
- $$\beta = \frac{f_0^2(1+\Delta)}{(1+\Delta-\rho\Delta)^2}.$$
- $$\alpha = \left[1 - \frac{f_0}{1+\Delta-\rho\Delta}\right]\left[\frac{f_0(1+\Delta)}{1+\Delta-\rho\Delta} - 1\right].$$

Clearly, since $\rho\Delta < 1$ (because Δ is at most $T_0/\tilde{c}_1 = 1/\rho$), for any value of f_0, both μ and β take on non-zero and therefore valid parameter values. Demanding $\alpha \geq 0$ sets bounds on the allowed values of f_0:

$$1 - \frac{\rho\Delta}{1+\Delta} \leq f_0 \leq 1 + \Delta - \rho\Delta \tag{18}$$

Often the second limit may be larger than 1 so that the effective range runs from $[1 - \rho\Delta/(1 + \Delta), 1]$ for f_0. For our data we typically find that the lower bound on f_0 is close to 1 and that, as a consequence, there is only a limited range in f_0, which in turn implies that we can accurately estimate α, β, and μ from our maximum likelihood fit.

References

[1] N. Q. Balaban, J. Merrin, R. Chait, L. Kowalik, and S. Leibler. Bacterial persistence as a phenotypic switch. *Science*, 305(5690):1622–1625, Sep 2004.
